# Effects of the Addition of Herbs on the Properties of Doenjang

**DOI:** 10.3390/foods10061307

**Published:** 2021-06-07

**Authors:** Sunmin Lee, Yang-Bong Lee, Choong-Hwan Lee, Inmyoung Park

**Affiliations:** 1 Department of Bioscience and Biotechnology, Konkuk University, Seoul 05029, Korea; duly123@naver.com; 2 Department of Food Science and Technology, Pukyong National University, Busan 48513, Korea; yblee@pknu.ac.kr; 3 School of Culinary Arts, Youngsan University, Busan 48015, Korea

**Keywords:** doenjang, herb, metabolomics, antioxidant activity, fermentation period

## Abstract

Three types of doenjang, a fermented soybean paste, were prepared by adding coriander (CR), Korean mint (KM), and peppermint (PM) and compared to the control group (CN) by studying their metabolite profiles and antioxidant activities followed by different fermentation periods (1, 30, and 150 days, respectively). The primary metabolome was analyzed by GC-TOF-MS, and 36 of metabolites were identified in four types of doenjang samples (CN, CR, KM, and PM). Samples were clustered based on the herb type and fermentation period in PCA and PLS-DA analysis. For the secondary metabolome analysis, UHPLC-Q-orbitrap-MS was used, and 26 metabolites were identified. The statistical analysis showed that the samples were clustered by herb type rather than fermentation period, and the samples containing KM and PM were located in the same group. The DPPH assay showed that PM-containing doenjang had the highest antioxidant activity. Correlation analysis indicated that organic acids such as lactic acid, malonic acid, succinic acid, uracil, vanillic acid, and quinic acid showed positive correlation with the DPPH activity. Overall, our results demonstrated that incorporating herbs in doenjang during fermentation caused significant shifts (*p*-value < 0.05) in the doenjang metabolites and antioxidant activity. Hence, herbs could be utilized for enhancing doenjang fermentation.

## 1. Introduction

Fermented food is an essential part of traditional diets in many cultures. Doenjang, a fermented soybean paste, is a traditional food that has a long culinary history in Korea. Diverse microorganisms are involved in the fermentation of doenjang and produce its unique flavors by decomposing the soybean proteins. Doenjang is rich in amino acids, fatty acids, organic acids, sugars, and isoflavones [1,2]. Numerous studies have investigated and described the broad range of biological activities of doenjang, including antioxidant, antimutagenic, anti-inflammatory, antimicrobial, antidiabetic, anticholesterol, anticancer, and antigenotoxic effects [3,4,5].

Traditionally, doenjang is made from meju, a fermented solid block of crushed and cooked soybeans prepared using natural flora [6]. The nutrient value, taste, and texture of doenjang considerably depend on the fermentation conditions, basic ingredients, and involvement of microorganisms [7,8]. Members of the *Bacillus* genus such as *B. subtilis*, *B. licheniformis*, and *B. megaterium* are the dominant bacteria and members of the *Aspergillus*, *Mucor*, and *Rhizopus* genera are the dominant fungi in the fermentation process [9,10,11]. Note that fermented soybean foods are significantly affected by the type and variety of microorganisms used for inoculation during fermentation, which determines their characteristic taste and flavor. Among the several factors, the environmental variables and the basic ingredients mainly affect the microbial composition of doenjang during fermentation [12].

In recent times, to improve the functional and sensorial qualities, doenjang has been prepared with a short-term fermentation method using *meju* supplemented with medicinal herbs and plant extracts [13,14].

Herbs and spices have long been used in the Korean cuisine for improving sensory properties such as flavoring and masking the off-flavor, increasing the shelf-life of foods, as well as for various pharmaceutical uses because herbs and spices contain large amounts of phenolic compounds and have an antimicrobial effect [15,16]. *Coriander* (*Coriandrum sativum*) is an annual herbaceous plant and a member of the Umbelliferae family. It is widely cultivated in Asia, North Africa, and Central Europe. Its green leaf is low in cholesterol and a rich source of iron, minerals, and vitamins [15,17]. In addition, numerous clinical studies have described the broad range of the biological activities of coriander, including antioxidant, antimicrobial, anti-inflammatory, and anticancer activities [18,19,20]. Korean mint (KM) (*Agastache rugosa*) is a traditional medicinal and ornamental plant and a member of the Lamiaceae family. It is mainly distributed in East Asia and is used as a spice. Traditionally, Korean mint is used for the treatment of anorexia, vomiting, and miasma. In addition, Korean mint has various physiological properties, including anticancer, antimicrobial, antipyretic, and antiviral activities [21,22]. Peppermint (*Mentha canadensis* L.) is a medicinal and aromatic herb belonging to the *Lamiaceae* family it has been reported to show antimicrobial, antioxidant, and anticancer activity [16,23]. We chose coriander, Korean mint, and peppermint owing to their antimicrobial properties, and because they are widely used in the cuisines of South Asia, Korea, and the West. Due to the antimicrobial properties of the herbs, the microbial environment of the soybean paste with herbs changes during the fermentation period [22,23,24], which in turn affects the metabolomic profiles and antioxidant properties of the paste.

In recent years, metabolomic analysis has been used to compensate for the drawbacks of chromatographic analytical techniques to obtain high-throughput measurements of the metabolites present in cells, tissues, and biofluids. As metabolomics enables the comprehensive analysis of metabolites, it is a valuable tool in food science for food quality assessment and component analysis [25,26]. Although many studies have investigated the production of commercially modified doenjang, few have explored the preparation of modified doenjang by adding herbs into salt solution along with meju during fermentation. This study aims to develop a modified fermentation environment of doenjang by adding coriander, Korean mint, and peppermint, which are known for their antimicrobial properties and consequently affect the doenjang’s quality. Thus, distinct metabolites of the modified doenjang were analyzed by gas chromatography–time-of-flight mass spectrometry (GC-TOF-MS) for primary metabolites and ultra-high-performance liquid chromatography–linear trap quadrupole–orbitrap-tandem mass spectrometry (UHPLC-LTQ-orbitrap-MS/MS) for secondary metabolites. Additionally, the antioxidant activity was evaluated by a DPPH assay.

## 2. Materials and Methods 

### 2.1. Preparation of Doenjang Samples

Fermented doenjang was prepared using the traditional Korean method under the guidance of a master craftsman of Korean cuisine. Briefly, fermented doenjang–meju bricks (approximately 2 kg, Gijang County, Korea) were soaked in 6 L of 20% (*w*/*v*) solar salt (Shinan County, Korea) solution in a porcelain pot. Next, three pieces of pure charcoal (3 cm × 3 cm × 10 cm) and five pieces of dried red pepper purchased from the local market (Gijang County, Korea) were added. Coriander, Korean mint, and peppermint were purchased from Samsung Welstory Food and Materials Distribution Headquarters (Seongnam-Si, Korea). The root of each herb was removed and trimmed. Then, herbs equal to 10% of the weight of the meju were added to the above solution. The resulting solution was stored in an open sunny yard with no temperature control, and the lid of the pot was opened by day and closed by night for 45 days. After 45 days of fermentation, the mixture was separated into liquid (soy sauce) and solid parts (doenjang). The solid part, which became doenjang, was mashed, homogenized, and continually stored under the same conditions and subjected to further fermentation. The doenjang samples containing each herb were prepared in triplicate. The samples were collected on days 1, 30, and 150 from the starting date of the fermentation for the analysis of metabolomes and antioxidants. The samples according to herbs and fermentation days are color expressed on Table 1.

### 2.2. Sample Preparation

The lyophilized doenjang samples (100 mg) were extracted with 1 mL of 80% methanol solution in a Mixer Ball Mill at 30 Hz/s for 10 min, followed by centrifugation at 10,000× *g* for 10 min at 4 °C. This process was repeated twice, then, 1 mL of supernatant was dried using a speed vacuum concentrator. The residues were redissolved in 500 µL of 80% methanol and filtered through a 0.22 µm filter for UHPLC-orbitrap-LTQ-IT-MS/MS and DPPH assays. For GC-TOF-MS, 100 µL of filtered samples were again dried using a speed vacuum concentrator prior to a two-stage derivatization step. Derivatization was performed by the method adapted from Bajpai et al. with some modifications [27]. First, oximation analysis was performed by dissolving the re-dried sample extracts with 50 μL of methoxyamine hydrochloride in pyridine (20 mg/mL) and incubating the reaction mixture at 30 °C for 90 min. Next, silylation was carried out by adding 50 µL of *N*-methyl-*N*-(trimethylsilyl)trifluoroacetamide (MSTFA) and reaction incubation was performed at 37 °C for 30 min.

### 2.3. GC-TOF-MS Analysis

GC-TOF-MS analysis for doenjang sample extracts was carried out using an Agilent 7890A GC system (Agilent Technologies, Santa Clara, CA, USA) coupled with the Pegasus HT TOF-MS (Leco Corporation, St. Joseph, MI, USA) and an Agilent 7693 autosampler. The samples were separated on an Agilent Rtx-5MS column (30 m length × 0.25 mm i.d. × 0.25 µm film thickness; Restk Corp., Bellefonte, PA, USA). The operational parameters were adapted from Lee et al. [10].

### 2.4. UHPLC-Q-orbitrap-MS/MS Analysis

UHPLC-Q-orbitrap-MS/MS analysis for doenjang sample extracts was performed using a heated electrospray ionization source (Thermo Fisher Scientific, Waltham, MA, USA), equipped with a Dionex UltiMate 3000 UHPLC system (Ultimate 3000 RS pump, Ultimate 3000 RS column compartment, and Ultimate 3000 RS autosampler; Dionex Corporation, CA, USA). The samples were separated on a hypersil gold C18 selectivity LC column (1.9 μm internal diameter, 50 mm × 2.1 mm; Thermo Fisher Scientific, Waltham, MA, USA). The operational parameters were adapted from Lee et al. [10].

### 2.5. DPPH Assay

The DPPH assay was performed with the method adapted from Dietz et al. with some modifications [28,29]. For the DPPH assay, the doenjang extract (20 μL), which has been described in Section 2.2, was mixed with the DPPH solution (180 μL, 0.2 mM in ethanol) in a 96-well plate, followed by incubation for 20 min at room temperature in the dark. The absorbance of the samples, which indicates the concentrations of DPPH free radicals, was measured at 515 nm using a spectrophotometer.

### 2.6. Data Processing and Multivariate Statistical Analysis

The raw data files from GC-TOF-MS and UHPLC-Q-orbitrap-MS/MS were converted into the computable document form (.cdf) format using LECO Chroma TOF software v.4.44 (Leco Co., CA, USA) and Thermo Xcalibur v.2.2 (Thermo Fisher Scientific), respectively. The converted data files were processed using the MetAlign software package (http://www.metalign.nl, accessed on 4 October 2012) to obtain a data matrix of accurate masses (*m*/*z*), normalized peak intensities, and retention times. Multivariate statistical analyses were performed using SIMCA-P^+^ 12.0 software (Umerics, Umea, Sweden) to analyze the differences among the metabolomics data of the fermented doenjang samples. Both unsupervised principal component analysis (PCA) and supervised partial least squares discriminant analysis (PLS-DA) were performed on the metabolomic datasets. The metabolites were identified by comparing their retention time and mass fragment patterns with the standard compounds, in-house library data, National Institute of Standards and Technology (NIST) database (version 2.0, 2011; Gaithersburg, MD, USA), and references. Samples with significantly different metabolites from other samples were selected with variable importance in projection (VIP) value > 0.7 and *p*-value < 0.05. The significant differences (*p*-value < 0.05) were evaluated by one-way ANOVA using STATISTICA 7 (Sta Soft Inc., Tulsa, OK, USA). In the DPPH assay, significant differences were tested by ANOVA followed by Duncan’s multiple range test using PASW Statistica 18 (SPAA, Inc., Chicago, IL, USA).

## 3. Results and Discussion

### 3.1. Effects of Different Herbs on the Primary Metabolomes of Doenjang

**Figure 1:**
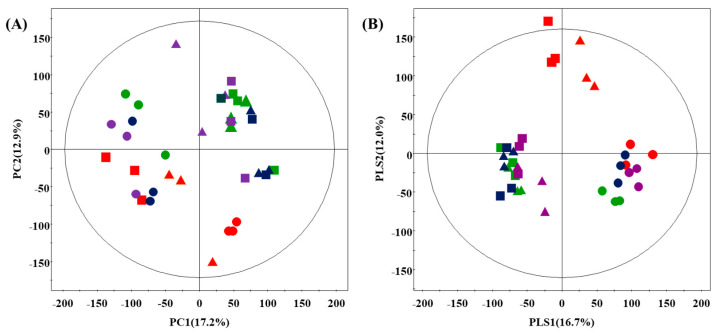
Principal component analysis (PCA) (**A**) and partial least squares discriminative analysis (PLS-DA) (**B**) score plot of the primary metabolome of doenjang with different herbs derived from GC-TOF-MS. Red, D_CN; green, D_CR; blue, D_KM; purple, D_PM; ●, day 1; ▲, day 30; ■, day 150.

To investigate the effects of coriander, Korean mint, and peppermint on the primary metabolome of doenjang, metabolite profiling was performed using GC-TOF-MS, followed by multivariate statistical analysis of the corresponding datasets. PCA and PLS-DA datasets showed that the herbs change the metabolite distribution of doenjang (Figure 1). The doenjang samples can be grouped into four types, D_CN (control doenjang), D_CR (doenjang with coriander), D_KM (doenjang with Korean mint), and D_PM (doenjang with peppermint), based on the fermentation period and herb type, with a total variability of 30.1% (PC1, 17.2% PC2, 12.9%) and 28.7% (PLS1, 16.7% PLS2, 12.0%), respectively. The primary metabolites were grouped based on the fermentation period and herb type using PLS-DA analysis (Figure 1B).

All day 1 samples were grouped together. Samples containing coriander, Korean mint, and peppermint were grouped on day 30 and day 150, except for control samples, indicating that each herb affected the primary metabolite as the fermentation progressed. To select the significant primary metabolites present in the doenjang samples using the PLS-DA model, the (VIP) value (>0.7) and *p-*value (<0.05) were determined from the GC-TOF-MS dataset [29]. These metabolites were subsequently identified using the standard compounds followed by comparing the resulting mass fragmentation patterns with the in-house *library* (Supplementary Table S1). They were then cross-verified using the published literature [30,31]. Based on the PLS1 and PLS2 components, 36 significant discriminant primary metabolites were identified for doenjang (Table 2). Overall, nine organic acids (lactic acid, glycolic acid, malonic acid, succinic acid, fumaric acid, malic acid, vanillic acid, citric acid, and quinic acid), 12 amino acids (alanine, isoleucine, valine, leucine, proline, serine, threonine, pyroglutamic acid, GABA, phenylalanine, and tyrosine), eight sugars and sugar alcohols (xylose, lyxose, fructose, galactose, N-acetyl-D-glucosamine, lactose, maltose, and myo-inositol), five fatty acids (palmitic acid, linoleic acid, oleic acid, linolenic acid, and stearic acid), and two nucleotides (uracil and cytosine) were identified.

The peak area of the identified metabolites was represented by the fold change obtained following normalization to the average for each metabolite (Table 2). Briefly, we obtained the mean relative area of each metabolite through multivariate analysis. We observed a common trend, depending on the fermentation period, among the metabolic datasets of the extracts of doenjang with the three herbs. In all the four groups of doenjang, the concentrations of lactic acid, malonic acid, vanillic acid, most amino acids (alanine, isoleucine, valine, leucine, isoleucine, proline, serine, threonine, and pyroglutamic acid), myo-inositol, and fatty acids increased with the fermentation period, whereas those of fumaric acid, malic acid, citric acid, and most sugars (xylose, lyxose, fructose, galactose, *N*-acetyl-*D*-glucosamine, lactose, and maltose) decreased. As shown in Table 2, the addition of herbs exerted a significant effect on the primary metabolome of doenjang. Further, doenjang made with peppermint had a higher concentration of organic acids than that made with coriander and Korean mint. Notably, doenjang prepared with herbs had a higher concentration of fatty acids than did the control doenjang. Our results revealed that the use of herbs, including coriander, Korean mint, and peppermint, may increase the concentration of primary metabolites in doenjang by natural fermentation. Furthermore, the concentration of the primary metabolome of doenjang may change depending on the fermentation period [8]. The difference in the chemical composition of the herbs may be responsible for the change in the primary metabolome of doenjang made with herbs [32].

### 3.2. Effects of the Different Herbs on the Secondary Metabolome of Doenjang

**Figure 2:**
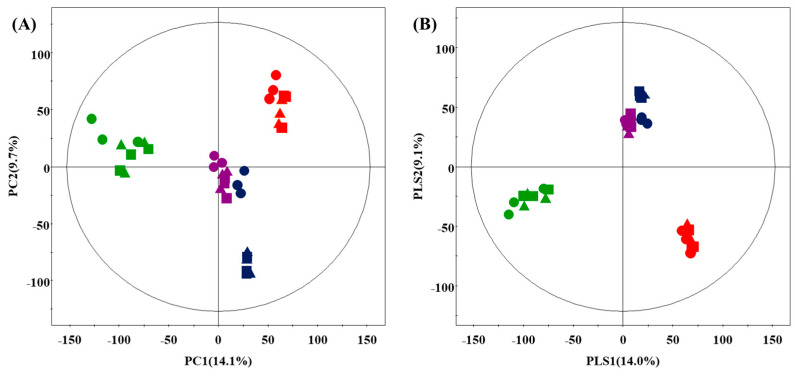
PCA (**A**) and PLS-DA (**B**) score plot of the secondary metabolome of doenjang with different herbs derived from UHPLC-Q-orbitrap-MS. Red, D_CN; green, D_CR; blue, D_KM; purple, D_PM; ●, day 1; ▲, day 30; ■, day 150.

To investigate the effects of coriander, Korean mint, and peppermint on the doenjang primary metabolome, metabolite profiling was performed using UHPLC-Q-orbitrap-MS, followed by multivariate statistical analysis of the corresponding datasets. PCA and PLS-DA datasets derived from UHPLC-Q-orbitrap-MS showed that the herbs changed the metabolite distribution in doenjang (Figure 2). Four types of doenjang, D_CN, D_CR, D_KM, and D_PM, were mainly clustered according to the herb type, with a total variability of 23.8% (PC1, 14.1% PC2, 9.7%) and 23.1% (PLS1, 14.0% PLS2, 9.1%). In particular, the doenjang samples containing peppermint and Korean mint were grouped together based on the PLS-DA analysis results (Figure 2B).

To select the significant secondary metabolites present in the doenjang samples using the PLS-DA model, the VIP value (>0.7) and *p*-value (<0.05) were determined from the UHPLC-Q-orbitrap-MS datasets. The discriminated metabolites from the UHPLC-Q-orbitrap-MS data were subsequently identified using the standard compounds followed by comparing the resultant mass fragmentation patterns with the in-house library (Supplementary Table S2). They were then cross-verified using the published literature [30,31]. A total of 26 significant discriminant secondary metabolites were selected on the basis of the PLS1 and PLS2 components (Table 3). Overall, 3 isoflavones (acetylgenistin, glycitein, and genistein), 16 soyasaponins (tetra-deacetyl-soyasaponin Ab, di-deacetyl-soyasaponin Ab, deacetyl-soyasaponin Ab, soyasaponin Bd, soyasaponin Bf, soyasaponin Ab (A1), soyasaponin Ac, soyasaponin Af (A2), soyasaponin Ba (V), soyasaponin Bb (I), soyasaponin Bc (II), soyasaponin Bb’ (III), soyasaponin Bc’ (IV), soyasaponin Bb-DDMP (βg), soyasaponin γg, and soyasaponin Bc-DDMP (βa)), and 7 lysophospholipids (lysoPC18:3, lysoPC16:1, lysoPC18:2, lysoPE16:0, lysoPC16:0, lysoPC18:1, and lysoPC18:0) were identified.

The peak area of the identified metabolites was represented by the fold change obtained following normalization to the average for each metabolite. We observed a common trend, depending on the fermentation period, among the metabolic datasets of the doenjang extracts with the three herbs. As shown in Table 3, the addition of herbs exerted a significant effect on the secondary metabolome of doenjang. Further, such doenjang samples showed a higher concentration of secondary metabolites, including isoflavones, soyasaponins, and lysophospholipids, than those made without herbs (control). Notably, doenjang made with Korean mint and peppermint showed a higher concentration of soyasaponins and lysophospholipids than that made with coriander. These results revealed that doenjang made with herbs, including coriander, Korean mint, and peppermint, may have higher concentrations of secondary metabolites, perhaps owing to the difference in the chemical composition of the herbs [20]. In contrast, the concentrations of acetylgenistin and most of soyasaponins and lysophospholipids decreased with an increase in the fermentation period.

### 3.3. Correlation between Bioactivities and Significant Discriminant Metabolites

**Figure 3:**
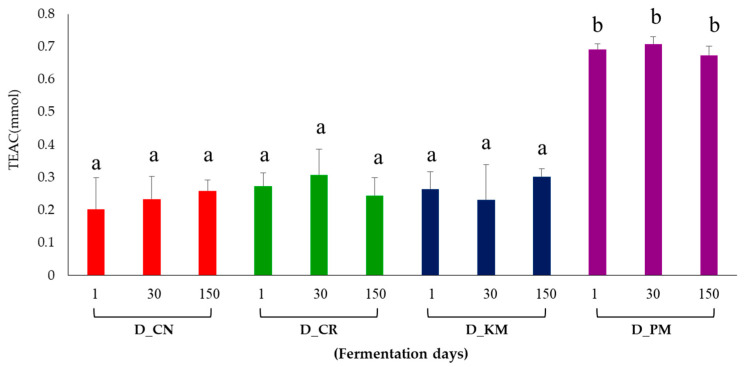
Comparison of the antioxidant activity (DPPH) of doenjang with different herbs. Red, D_CN; green, D_CR; blue, D_KM; purple, D_PM. Different letters in the bar graph indicate the significant differences obtained by Duncan’s multiple range test (*p*-value < 0.05).

Considering the varying impact of the herbs on doenjang metabolites, we analyzed the antioxidant activity of doenjang based on the fermentation period using the DPPH assay (Figure 3). Different antioxidant activities were observed depending on the type of herb added, not on the fermentation period. Korean mint and peppermint contain different flavonoids (tilianin, acacetin, linarin, and agastachoside), terpenes, pulegone, estragole, p-Menthan-3-one, and monoterpenes [33], while coriander contains significant amounts of flavonoids, phenolic acids, polyphenols, and anthocyanins [32]. As shown in Figure 3, the doenjang samples show different antioxidant activities, possibly owing to the difference in the chemical composition of the herbs. Peppermint contains higher concentrations of flavonoids and phenolic compounds than Korean mint and coriander [32,33,34]. Our results revealed that the addition of herbs improved the functionality, flavor, and antioxidant and biological activities of doenjang.

**Figure 4:**
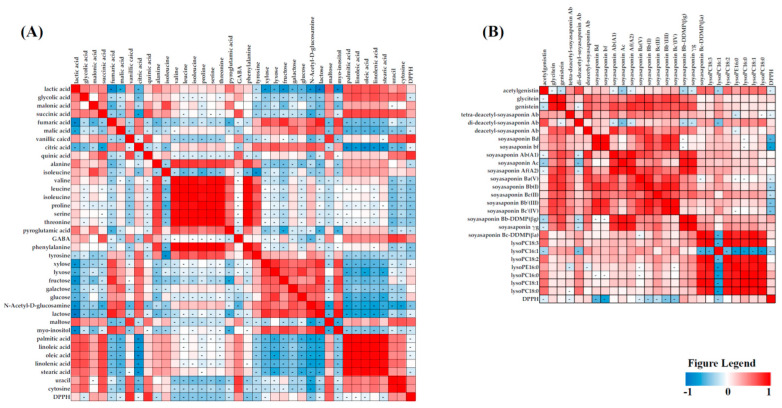
Correlation map between the metabolites (primary (**A**) and secondary (**B**)) and antioxidant activity.

We performed a Pearson correlation analysis to confirm the relationship between the metabolite composition and antioxidant activity through a correlation assay (Figure 4) [35]. Di-deacetyl-soyasaponin Ab, glycitein, deacetyl-soyasaponin Ab, soyasaponin Ac, soyasaponin Bb-DDMP(βg), soyasaponin γg, lysoPC 16:0, lactic acid, isoleucine, malonic acid, succinic acid, uracil, GABA, cytosine, vanillic acid, quinic acid, and myo-inositol showed a positive correlation with the DPPH activity. The correlation analysis also revealed that the bioactivity of doenjang varies according to the relation between the bioactivity value and the metabolite content of the doenjang samples [36]. These results suggest that the addition of herbs influences the antioxidant activity of doenjang.

## 4. Conclusions

Metabolomics analysis performed with doenjang prepared by adding three different herbs (coriander, Korean mint, and peppermint) revealed significant shifts in the metabolite composition. The primary metabolites were affected by the herb type and fermentation period, while the secondary metabolites were more affected by the herb type, perhaps because of bioactive compounds of the herbs. Consequently, doenjang containing herbs showed positive changes in the metabolite composition with an increase in isoflavones, soyasaponins, and lysophospholipids, and a higher antioxidant capacity than doenjang made without herbs. Further studies should be conducted to investigate the effects of adding herbs on doenjang for a longer fermentation period by conducting a volatile compound analysis for flavor and a sensory evaluation such as a consumer preference test of doenjang containing herbs.

## Figures and Tables

**Table 1:** Color expression according to the fermentation period of doenjang, including general doenjang and when three different herbs were added during fermentation.

Fermentation Period (Days)	Doenjang (with Herbs)			
	Control	Coriander	Korean Mint	Peppermint
	D_CN	D_CR *	D_KM	D_PM
Day 1	●	●	●	●
Day 30	▲	▲	▲	▲
Day 150	■	■	■	■

* CN, control; CR, Coriandrum sativum; KM, Agastache rugosa; PM, Mentha canadensis.

**Table 2:** Relative values (peak areas) of the primary metabolites analyzed by GC-TOF-MS in doenjang made with three different herbs ^1^.

	D_CN			D_CR			D_KM			D_PM		
Fermentation Period (Days)	1	30	150	1	30	150	1	30	150	1	30	150
*Organic acids*												
Lactic acid	0.65	1.04	1.08	0.78	1.18	1.18	0.76	1.16	1.05	0.84	1.12	1.17
Glycolic acid	0.44	0.88	0.99	1.03	1.39	1.42	1.16	1.08	0.94	0.80	0.77	1.11
Malonic acid	0.50	1.01	1.26	0.40	0.61	0.64	0.77	1.08	1.78	0.73	1.34	1.86
Succinic acid	0.46	0.63	0.64	0.97	1.03	1.12	1.05	1.32	1.33	1.04	0.99	1.43
Fumaric acid	2.04	0.95	0.54	2.41	0.80	0.57	1.43	0.42	0.55	1.58	0.27	0.45
Malic acid	1.74	1.36	1.45	0.71	0.20	0.51	2.77	0.38	0.49	1.85	0.46	0.08
Vanillic acid	0.64	0.86	0.82	0.80	1.02	1.08	0.60	0.68	0.66	1.42	1.50	1.92
Citric acid	2.25	2.93	1.59	1.05	0.07	0.10	0.85	0.09	0.08	2.65	0.11	0.23
Quinic acid	0.28	0.31	0.29	0.60	0.61	0.67	1.04	1.33	1.06	2.00	1.56	2.25
*Amino acids*												
Alanine	0.79	1.13	1.13	0.74	0.86	1.24	0.83	1.42	1.14	0.86	0.71	1.14
Valine	0.84	1.31	1.37	0.67	0.58	0.94	0.99	1.62	1.27	0.79	0.66	0.97
Leucine	0.99	1.28	1.30	0.77	0.54	1.04	1.02	1.45	1.14	0.93	0.61	0.93
Isoleucine	0.94	1.27	1.30	0.73	0.57	0.97	1.04	1.48	1.20	0.85	0.71	0.93
Proline	0.92	1.50	1.54	0.68	0.37	0.95	1.04	1.86	1.04	0.98	0.36	0.76
Serine	0.91	1.46	1.50	0.51	0.44	0.95	1.03	1.93	1.11	0.86	0.45	0.86
Threonine	0.99	1.46	1.50	0.53	0.42	0.87	1.03	1.77	1.15	0.88	0.48	0.91
Pyroglutamic acid	0.58	1.64	1.91	0.41	0.81	0.97	0.51	1.35	1.35	0.49	0.74	1.25
GABA	0.42	0.54	0.58	1.22	0.86	1.47	0.82	1.23	1.07	1.55	0.90	1.35
Phenylalanine	0.91	1.33	1.39	0.73	0.49	1.04	0.99	1.70	1.15	0.91	0.48	0.89
Tyrosine	1.31	1.39	1.27	0.94	0.55	1.13	1.01	1.17	0.77	1.36	0.20	0.90
*Sugars and sugar alcohols*												
Xylose	2.44	1.31	0.11	1.65	0.23	0.15	2.32	0.24	0.35	2.16	0.10	0.94
Lyxose	1.50	1.51	0.78	1.20	0.61	0.47	1.36	0.30	0.51	1.91	0.60	1.24
Fructose	1.94	0.41	0.07	3.32	0.04	0.06	1.56	0.01	0.10	4.14	0.15	0.19
Galactose	0.98	1.50	1.01	1.05	0.43	0.40	2.04	0.58	0.90	1.71	0.63	0.75
Glucose	1.21	1.24	0.95	1.03	0.77	0.59	1.30	0.69	0.88	1.15	1.03	1.16
*N*-Acetyl-*D*-glucosamine	1.90	1.53	1.31	1.19	0.42	0.43	1.96	0.65	0.72	1.04	0.42	0.43
Lactose	2.57	0.86	0.56	1.98	0.15	0.15	2.51	0.28	0.27	2.03	0.30	0.34
Maltose	1.50	0.77	0.42	1.98	0.36	0.41	2.65	0.88	0.61	1.73	0.36	0.32
Myo-inositol	0.53	0.96	0.98	0.67	1.20	1.27	0.56	0.87	0.74	1.23	1.22	1.76
*Fatty acids*												
Palmitic acid	0.63	0.79	0.92	1.04	1.17	1.21	0.94	1.14	1.28	0.80	0.97	1.10
Linoleic acid	0.78	0.91	0.98	1.02	1.09	1.10	0.98	1.06	1.12	0.89	0.98	1.08
Oleic acid	0.54	0.74	0.92	1.03	1.20	1.24	0.97	1.20	1.37	0.71	0.98	1.11
Linolenic acid	0.65	0.84	0.96	1.03	1.17	1.19	0.92	1.08	1.20	0.83	0.97	1.15
Stearic acid	0.59	0.73	0.90	1.01	1.18	1.27	0.92	1.25	1.46	0.73	0.88	1.08
*Nucleotides*												
Uracil	0.40	0.45	0.46	1.25	1.38	1.60	0.87	0.87	0.79	1.33	1.08	1.53
Cytosine	0.28	0.46	0.50	1.01	1.43	1.70	0.87	0.98	0.73	1.20	1.22	1.63

^1^ CN, control; CR, coriander; KM, Korean mint; PM, peppermint. The color values (blue to red) represent the fold change of each metabolite (blue indicates smallest and red indicates largest).

**Table 3:** Relative values of the secondary metabolites analyzed by UHPLC-Q-orbitrap-MS in doenjang with three different herbs ^1^.

	D_CN			D_CR			D_KM			D_PM		
Fermentation Period (Days)	1	30	150	1	30	150	1	30	150	1	30	150
*Isoflavones*												
Acetylgenistin	1.46	0.47	0.32	2.56	1.11	1.22	1.65	0.51	0.18	1.70	0.42	0.39
Glycitein	1.06	1.03	1.07	0.95	1.01	0.92	0.96	0.96	0.93	1.04	0.99	1.09
Genistein	1.07	1.07	1.08	0.93	0.95	0.87	0.98	1.01	0.99	1.02	0.98	1.04
*Soyasaponins*												
Tetra-deacetyl-soyasaponin Ab	1.50	1.26	1.22	1.15	0.93	0.95	1.04	0.48	0.53	1.21	0.80	0.92
Di-deacetyl-soyasaponin Ab	0.93	0.72	0.74	1.38	1.22	1.04	1.21	0.88	0.78	1.16	0.98	0.97
Deacetyl-soyasaponin Ab	1.28	1.35	1.11	1.04	0.90	0.81	0.87	0.67	0.66	1.20	1.09	1.02
Soyasaponin Bd	1.07	1.01	1.01	1.10	1.02	0.97	1.01	1.03	1.05	0.93	0.86	0.94
Soyasaponin Bf	1.11	1.04	1.02	1.09	1.01	0.97	1.02	1.08	1.05	0.91	0.80	0.91
Soyasaponin Ab(A1)	1.28	1.50	1.16	0.83	0.68	0.63	1.18	0.82	0.78	1.10	1.08	0.96
Soyasaponin Ac	1.53	1.48	1.40	0.07	0.04	0.58	1.39	1.01	0.94	1.26	1.14	1.17
Soyasaponin Af(A2)	1.23	1.20	1.15	1.21	1.20	1.18	1.08	0.96	0.83	0.70	0.65	0.63
Soyasaponin Ba(V)	1.11	1.11	1.04	1.07	0.98	0.91	0.99	0.91	0.94	0.94	1.01	1.00
Soyasaponin Bb(I)	1.08	1.06	1.03	1.02	0.99	0.94	1.02	0.99	0.97	0.99	0.94	0.98
Soyasaponin Bc(II)	1.05	1.00	1.02	0.97	0.98	0.96	1.08	1.00	0.99	1.01	0.93	1.00
Soyasaponin Bb’(III)	1.10	1.02	1.04	1.05	1.04	0.95	1.04	1.01	0.96	0.96	0.87	0.96
Soyasaponin Bc’(IV)	1.13	1.02	1.08	1.07	1.06	0.97	0.97	0.97	0.93	0.97	0.86	0.95
Soyasaponin Bb-DDMP(βg)	1.27	1.34	1.22	0.36	0.37	0.34	1.34	0.92	0.86	1.39	1.29	1.32
Soyasaponin γg	1.37	1.27	1.32	0.35	0.38	0.30	1.61	1.00	0.79	1.35	1.13	1.14
Soyasaponin Bc-DDMP(βa)	1.28	0.57	0.02	1.15	0.15	0.05	3.22	1.39	0.22	3.57	0.22	0.17
*Lysophospholipids*												
LysoPC18:3	1.35	0.76	0.14	1.45	0.40	0.28	2.20	1.53	0.68	2.01	0.74	0.46
LysoPC16:1	2.09	2.07	1.95	0.75	1.18	0.98	0.00	0.00	1.20	0.00	0.91	0.87
LysoPC18:2	1.41	0.90	0.26	1.29	0.55	0.37	1.96	1.40	0.71	1.90	0.63	0.63
LysoPE16:0	0.95	0.63	0.28	1.04	0.60	0.56	1.73	1.72	1.02	1.83	0.79	0.86
LysoPC16:0	0.84	0.59	0.19	1.13	0.74	0.58	1.83	1.24	0.70	2.65	0.75	0.76
LysoPC18:1	1.10	0.68	0.19	1.20	0.60	0.37	2.17	1.32	0.60	2.65	0.55	0.56
LysoPC18:0	0.94	0.69	0.33	1.35	0.76	0.73	1.52	1.32	0.89	1.76	0.80	0.91

^1^ CN, control; CR, coriander; KM, Korean mint; PM, peppermint. The color values (blue to red) represent fold change of each metabolite (blue indicates smallest and red indicates largest).

## Data Availability

Data is contained within the article or supplementary material.

## Supplementary Materials

The following are available online at https://www.mdpi.com/article/10.3390/foods10061307/s1. Table S1. Discriminative primary metabolites analyzed by GC-TOF-MS in doenjang made with three different herbs. Table S2. Discriminative secondary metabolites analyzed by UHPLC-Q-orbitrap-MS in doenjang made with three different herbs.
