# Supplementary material for: Effects of the Addition of Herbs on the Properties of Doenjang
Source: Foods. 2021 Jun 7;10(6):1307. doi: 10.3390/foods10061307 (PMC8227189; doi:10.3390/foods10061307)
Supplement: Supplementary file 1 [file foods-10-01307-s001.zip › foods-1228565-supplementary.pdf]

Supplemenatary Table 1. Discriminative Primary Metabolites analysed by GC–TOF-MS in doenjang made with three different herbs.

Compounds[a]	RT (min)[b]	Ms fragment ions (m/z)	VIP1[c]	VIP2
Organic acids				
Lactic acid	4.77	73, 117, 147, 191, 75, 148, 190, 66, 133, 118, 74, 59	2.31	1.84
Glycolic acid	4.96	73, 147, 66, 75, 148, 59, 177, 74, 133	0.99	0.78
Malonic acid	6.38	70, 147, 73, 86, 75, 148, 66, 149, 74	1.12	0.93
Succinic acid	7.42	147, 73, 75, 55, 148, 56, 149, 74, 129	1.04	1.18
Fumaric acid	7.73	73, 99, 147, 241, 75, 255, 126, 100, 113, 256, 245	2.13	1.71
Malic acid	9.00	73, 147, 75, 55, 133, 74, 101, 233	1.75	1.39
Vanillic caicd	11.17	73, 75, 174, 267, 297, 74, 59, 103, 223, 86, 126, 147	0.54	0.48
Citric acid	11.57	73, 147, 75, 273, 74, 142, 59, 218, 174, 67, 133, 149, 148, 129, 265	1.85	1.74
Quinic acid	11.90	73, 147, 75, 345, 174, 204, 74, 255, 103, 191, 133	0.43	0.96
Amino acid				
Alanine	5.25	73, 116, 147, 117, 75, 59, 74, 100	0.74	0.78
Valine	6.47	73, 100, 218, 147, 75, 74, 59, 133	0.21	0.91
Leucine	7.03	73, 158, 102, 100, 159, 116, 74, 75	0.23	0.85
Isoleucine	7.25	73, 158, 100, 218, 147, 75, 74, 159, 59	0.09	0.84
Proline	7.29	73, 142, 147, 143, 74, 75, 59, 216	0.25	0.95
Serine	7.88	73, 204, 218, 100, 147, 75, 74, 188, 116	0.05	0.89
Threonine	8.13	73, 117, 57, 101, 219, 218, 147, 75, 100	0.07	0.98
Pyroglutamic acid	9.33	73, 156, 147, 157, 75, 74, 230, 59, 258	0.92	1.63
GABA	9.34	73, 156, 147, 174, 75, 157, 59, 86, 74, 100	0.23	0.81
Phenylalanine	10.15	73, 218, 192, 100, 147, 75, 74, 219, 91, 193	0.06	0.82
Tyrosine	12.38	218, 147, 100, 103, 219, 117, 319, 148, 133	1.14	1.12
Sugar & sugar alcohols				
Xylose	10.41	73, 103, 217, 147, 74, 75, 133, 307, 59	1.83	1.47
Lyxose	10.46	103, 73, 147, 217, 189, 133, 160, 117, 129	1.72	1.34
Fructose	12.07	73, 103, 217, 147, 74, 307, 89, 117	2.10	1.75
Galactose	12.14	73, 103, 147, 205, 319	1.67	1.33
Glucose	12.19	73. 103. 147. 205. 319. 364	1.41	1.12
N-Acetyl-D-glucosamine	13.37	73, 147, 129, 87, 75, 117, 202, 103, 74, 205	2.12	1.74
Lactose	16.85	73, 204, 147, 217, 103, 129, 205, 169, 117	2.39	1.90
Maltose	17.02	73, 147, 204, 103, 217, 129, 361, 75, 169	2.15	1.78
Myo-inositol	13.42	73, 147, 217, 191, 305, 129, 74, 133	1.34	1.04
Fatty acids				
Palmitic acid	12.96	117, 75, 73, 132, 129, 145, 55, 131, 118, 313	1.80	1.51
Linoleic acid	14.01	75, 73, 117, 129, 55, 67, 81, 95, 96, 79	1.73	1.39
Oleic acid	14.02	117, 129, 75, 73, 145, 55, 96, 354	1.82	1.50
Linolenic acid	14.05	75, 79, 73, 67, 93, 95, 129, 117, 81, 335	1.79	1.45
Stearic acid	14.14	117, 75, 73, 132, 129, 145, 55, 131, 341	1.74	1.46
Nucleotides				
Uracil	7.71	99, 73, 147, 241, 113, 255, 126, 100, 256	0.44	0.97
Cytosine	9.41	73, 84, 75, 156, 147, 56, 98, 174, 74, 254, 240	0.79	0.96

[a]Identified compounds: mass spectrum was consistent with those of standard compounds

[b]Retention time

[c]Variables were selected based on variable importance of projection (VIP1 or VIP2 > 0.7)

Supplemenatary Table 2. Discriminative Secondary Metabolites analysed by UHPLC–Q-orbitrap-MS in doenjang made with three different herbs.

Tentative identification[a]	RT (min)[b]	posi	nega	MW	Elemental composition [M+H]+	MS^n fragment pattern (m/z)	VIP1[c]	VIP2
Isoflavones								
Acetylgenistin	5.75	475	473	474	C23H23O11	475>457,379,313,270>242,215,152	1.31	0.97
Glycitein	5.91	285	283	284	C16H13O5	285>285,270>252,242,214,152	0.71	0.57
Genistein	6.43	271	269	270	C15H11O5	271>253,247,243,215>156,129,96	1.53	1.10
Soyasaponins								
Tetra-deacetyl-soyasaponin Ab	5.25	1270	1268	1269	C59H97O29	-	0.62	1.58
Di-deacetyl-soyasaponin Ab	5.70	1354	1352	1353	C63H101O31	-	1.69	1.24
Deacetyl-soyasaponin Ab	6.30	1396	1394	1395	C65H103O32	-	1.03	1.26
Soyasaponin Bd	6.49	958	956	957	C48H77O19	-	0.15	0.82
Soyasaponin Bf	6.58	928	926	927	C47H75O18	925>907,717,>567,511,471	0.07	0.89
Soyasaponin Ab(A1)	6.66	1438	1436	1437	C67H105O33	1436>1188>1026	1.91	1.46
Soyasaponin Ac	6.73	1422	1420	1421	C67H105O32	-	2.19	1.56
Soyasaponin Af	6.89	1276	1274	1275	C61H95O28	1273>1231,1189,749,605>473	2.08	1.55
Soyasaponin Ba(V)	7.08	960	958	959	C48H79O19	960>797, 599, 441 > 581, 423	0.59	0.93
Soyasaponin Bb(I)	7.21	944	942	943	C48H79O18	943>325,797,599>581,423,351	0.86	1.08
Soyasaponin Bc(II)	7.33	914	912	913	C47H77O17	911>893,765,457>437,371	0.93	0.68
Soyasaponin Bb'(III)	7.37	798	796	797	C42H69O14	797>779,599,423,365>203	0.19	0.90
Soyasaponin Bc'(IV)	7.47	768	766	767	C41H67O13	765>721,615,457	0.06	1.34
Soyasaponin Bb-DDMP(βg)	7.58	1070	1068	1069	C54H85O21	1068>968>822	2.20	1.66
Soyasaponin γg	7.84	924	922	923	C48H74O17	922>822,642>464,598,484	2.21	1.63
Soyasaponin Bc-DDMP(βa)	8.54	1040	1038	1039	C53H83O20	-	0.34	0.83
Lysophospholipids								
LysoPC18:3	8.14	518	516	517	C26H49NO7P	518>500>162	0.28	0.86
LysoPC16:1	8.24	494	538	495	C24H49NO7P	494>476	1.17	1.85
LysoPC18:2	8.54	520	518	519	C26H51NO7P	520>502>323,184	0.39	0.79
LysoPE16:0	8.72	454	452	453	C21H45NO7P	454>436>393	0.17	1.51
LysoPC16:0	8.83	540	496	497	C24H51NO7P	496>478>419,125,86	0.11	1.22
LysoPC18:1	9.05	522	520	521	C26H53NO7P	522>504>419,309	0.16	0.98
LysoPC18:0	9.76	524	522	523	C26H55NO7P	524>506>311	0.36	1.26

[a]Tentative identified metabolites

[b]Retention time

[c]Variables were selected based on variable importance of projection (VIP1 or VIP2 > 0.7)
